# Neurosurgical Challenges in Recurrent Adhesive Arachnoiditis After Spinal Anesthesia for Cesarean Delivery: Case Report and Literature Review

**DOI:** 10.7759/cureus.86379

**Published:** 2025-06-19

**Authors:** Walter Fagundes, Mariana Z Zottele, Mariana Cavalcanti, Yasmin P Silva

**Affiliations:** 1 Neurosurgery, Federal University of Espirito Santo, Vitória, BRA; 2 Neurosurgery, GeNeuro - International Research Group in Neuroscience, Vitória, BRA; 3 General Physician, GeNeuro - International Research Group in Neuroscience, Vitória, BRA; 4 General Medicine, Healthcare Institution of South Iceland, Selfoss, ISL

**Keywords:** adhesive arachnoiditis, arachnoid cyst, cesarean delivery, spinal anesthesia, spine surgery

## Abstract

Adhesive arachnoiditis (AA) is a rare, chronic inflammatory condition affecting the pia-arachnoid layers, characterized by fibrosis and adhesion formation. It leads to nerve root adhesion and spinal cord compression, often resulting in severe neurological impairment. Commonly associated etiologies include prior spinal surgeries, infections, subarachnoid hemorrhage, epidural anesthesia, myelography, contrast agents, chemical irritation, and, occasionally, idiopathic factors. Following the literature review, 12 cases of AA related to anesthesia and obstetric procedures were identified.

We report the case of a 39-year-old woman presenting with lower limb weakness and thoracic paresthesia three months after receiving spinal anesthesia during a cesarean section. Magnetic resonance imaging (MRI) revealed extensive spinal cord edema and an expanding cystic lesion causing spinal cord compression. Despite initial corticosteroid treatment, the patient developed severe iatrogenic Cushing syndrome. She underwent two thoracic decompression surgeries, including microsurgical AA resection and cyst fenestration, which resulted in transient improvement but eventual symptom recurrence and persistent disability.

AA should be considered a potential complication in patients who develop neurological symptoms after spinal anesthesia. Early diagnosis via MRI and prompt initiation of corticosteroid therapy, surgical spinal cord decompression, and cyst drainage are critical for optimal outcomes, although recurrences are common.

## Introduction

Adhesive arachnoiditis (AA) is a rare condition characterized by chronic inflammation and scarring of the arachnoid and pia mater, potentially leading to nerve root adhesions, spinal cord compression, and significant disability [1,2]. The clinical symptoms often include paraparesis, sensory deficits in the lower limbs, back pain, and bladder dysfunction, although some individuals remain asymptomatic [1-3]. Predisposing factors include spinal surgery, infections (e.g., tuberculosis, syphilis), spinal trauma, tumors, subarachnoid hemorrhage, contrast myelography, and neuraxial anesthesia [4,5]. Notably, epidural or spinal anesthesia can provoke AA due to direct nerve injury, contamination, or chemical irritation, followed by inflammation and fibrosis of the arachnoid membranes. [6,7].

In this report, we present a case of AA that developed after spinal anesthesia for a cesarean delivery, which underwent two spinal thoracic decompressions due to a recurrence of spinal cord compression. We will discuss the diagnostic challenges and management strategies associated with this condition.

## Case presentation

**Figure 1:**
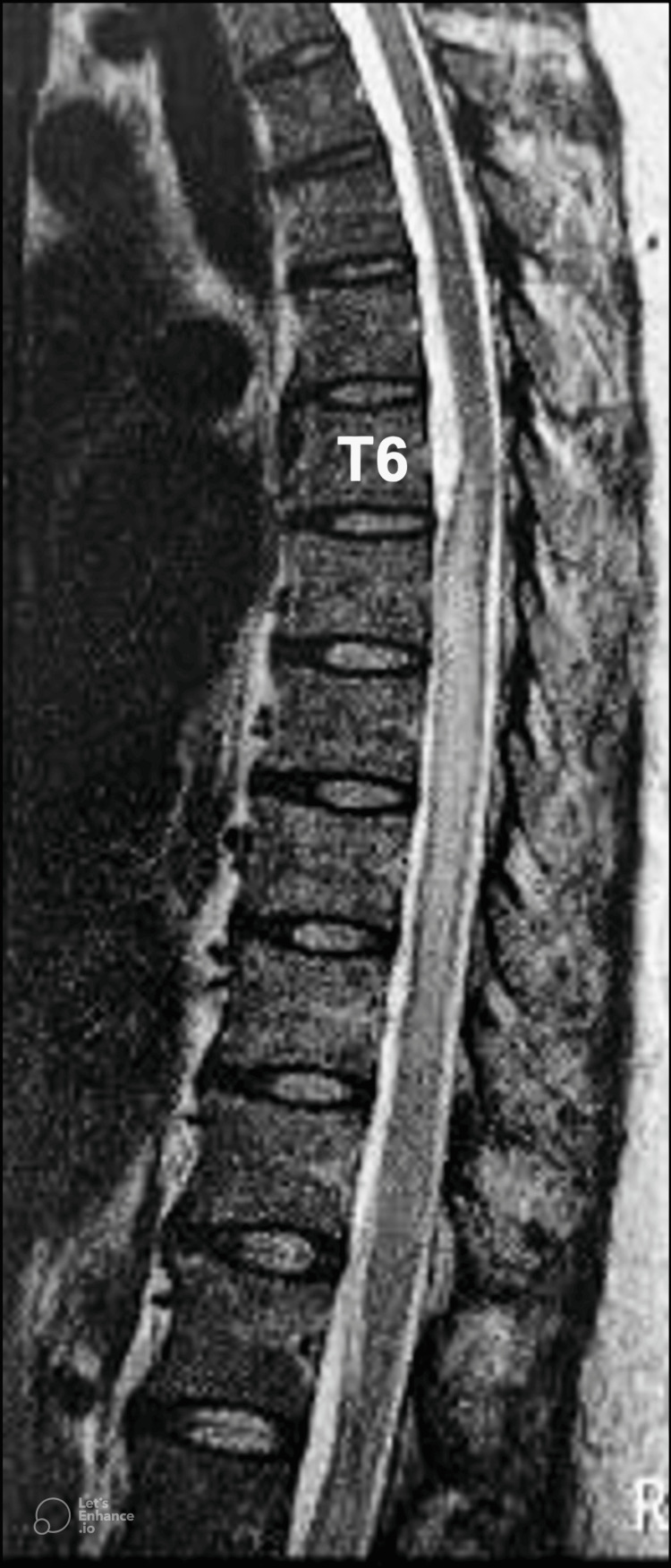
Sagittal T2-weighted MRI of the thoracolumbar spine three months after spinal anesthesia indicating edema of the spinal tissue and a cystic mass at the T5 level, causing mild compression of the dorsal column.

A 39-year-old woman underwent spinal anesthesia with 0.5% bupivacaine for a cesarean delivery. After several unsuccessful attempts at lumbar puncture for an intrathecal injection, she was administered general anesthesia. Four days later, the patient developed severe headaches that persisted for one month. Three months later, she returned with progressive bilateral lower limb weakness and sensory disturbances in the thoracic region. During the neurological examination, she presented with lower limb weakness (grade IV), and there was diminished sensation below the thoracic level. Magnetic resonance imaging (MRI) revealed spinal cord edema at the T6-T11 levels, with a cystic mass at the T5-T6 level, measuring 3.6 x 0.7 cm, causing mild dorsal compression (Figure 1).

Cervical and brain MRIs showed no abnormalities. Electromyography of the lower limbs was normal. Initial conservative management included prednisone (20 mg/day), physiotherapy, and acetazolamide. While there were initial improvements as she regained some light touch and pinprick sensation, improved paraparesis, and regained knee flexion, she soon developed prominent Cushingoid features, necessitating the withdrawal of corticosteroids.

**Figure 2:**
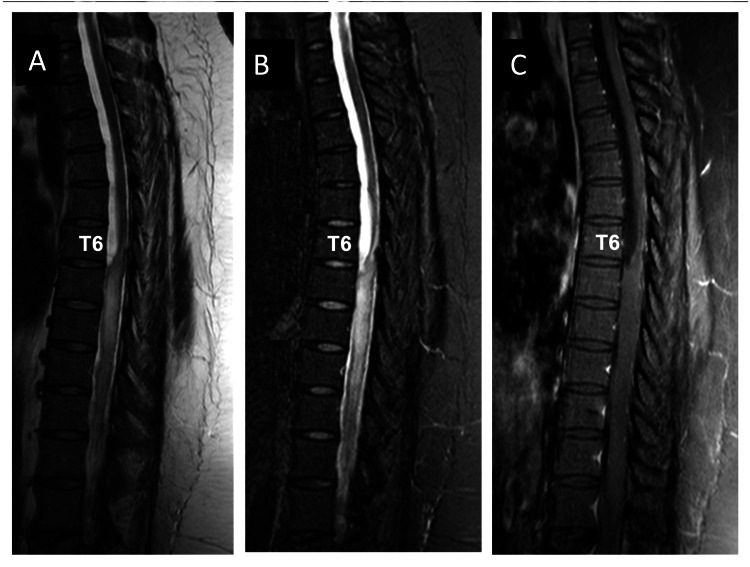
Sagittal T2-weighted (A), T2-STIR (B), and T1-weighted (C) MRI, one month before the first laminectomy, show a reduction of spinal cord edema (T7-T9 levels) and an increase in the cystic mass to the T5-T6 levels.

The T2-weighted (Figure 2A), T2-STIR sequence (Figure 2B), and T1-weighted (Figure 2C) MRI after eight months showed reduced T2 signal hyperintensity at the T7-T9 levels, but the cyst had increased in size (T5-T6), displacing the dorsal surface of the spinal cord. These changes were associated with returning to the initial clinical status.

**Figure 3:**
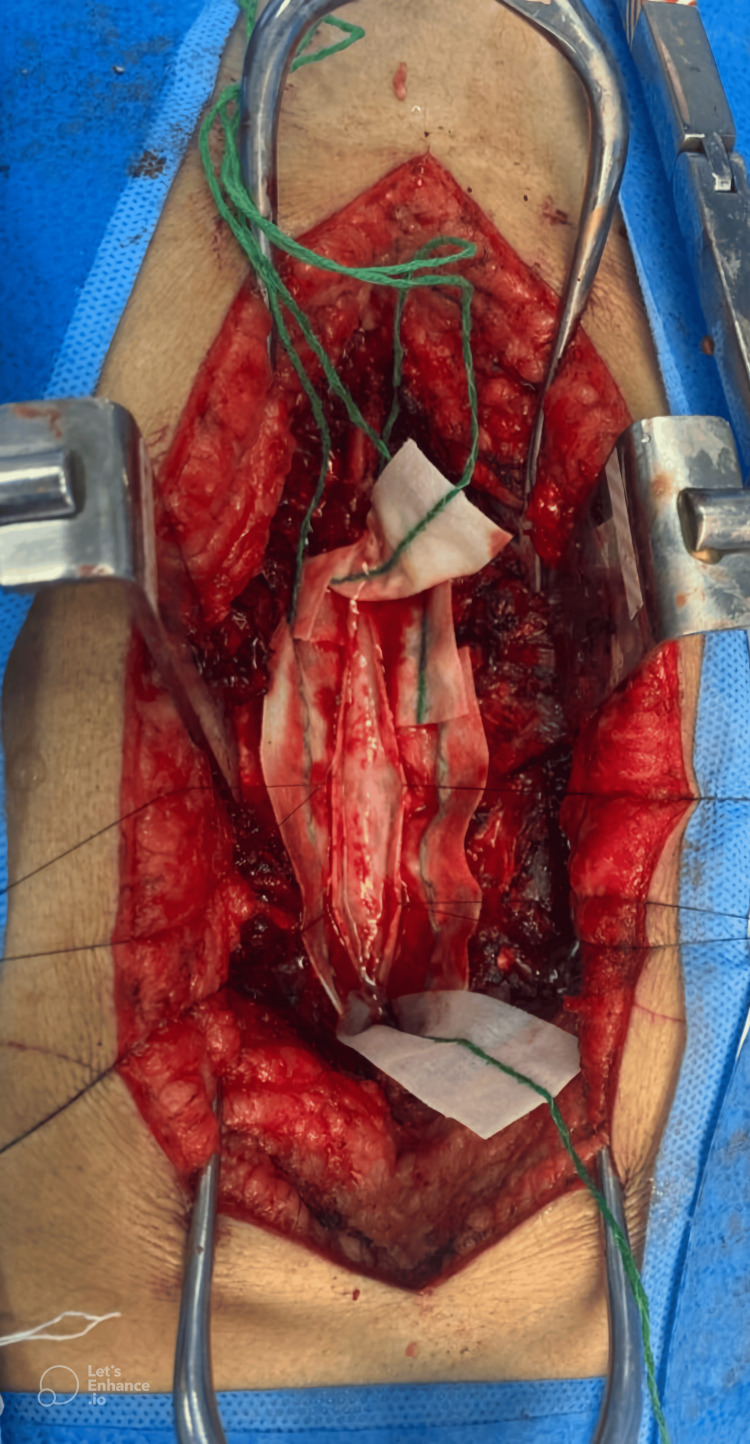
Intraoperative image showing the opened and retracted dura mater, and a dense fibrous pia-arachnoid meningeal lesion involving the spinal cord.

Due to ongoing neurological deterioration, the patient underwent a bilateral T3-T6 laminectomy with microsurgical resection of a dense fibrous pia-arachnoid meningeal lesion involving the spinal cord and nerve roots, which caused significant compression, followed by cyst fenestration (Figure 3).

**Figure 4:**
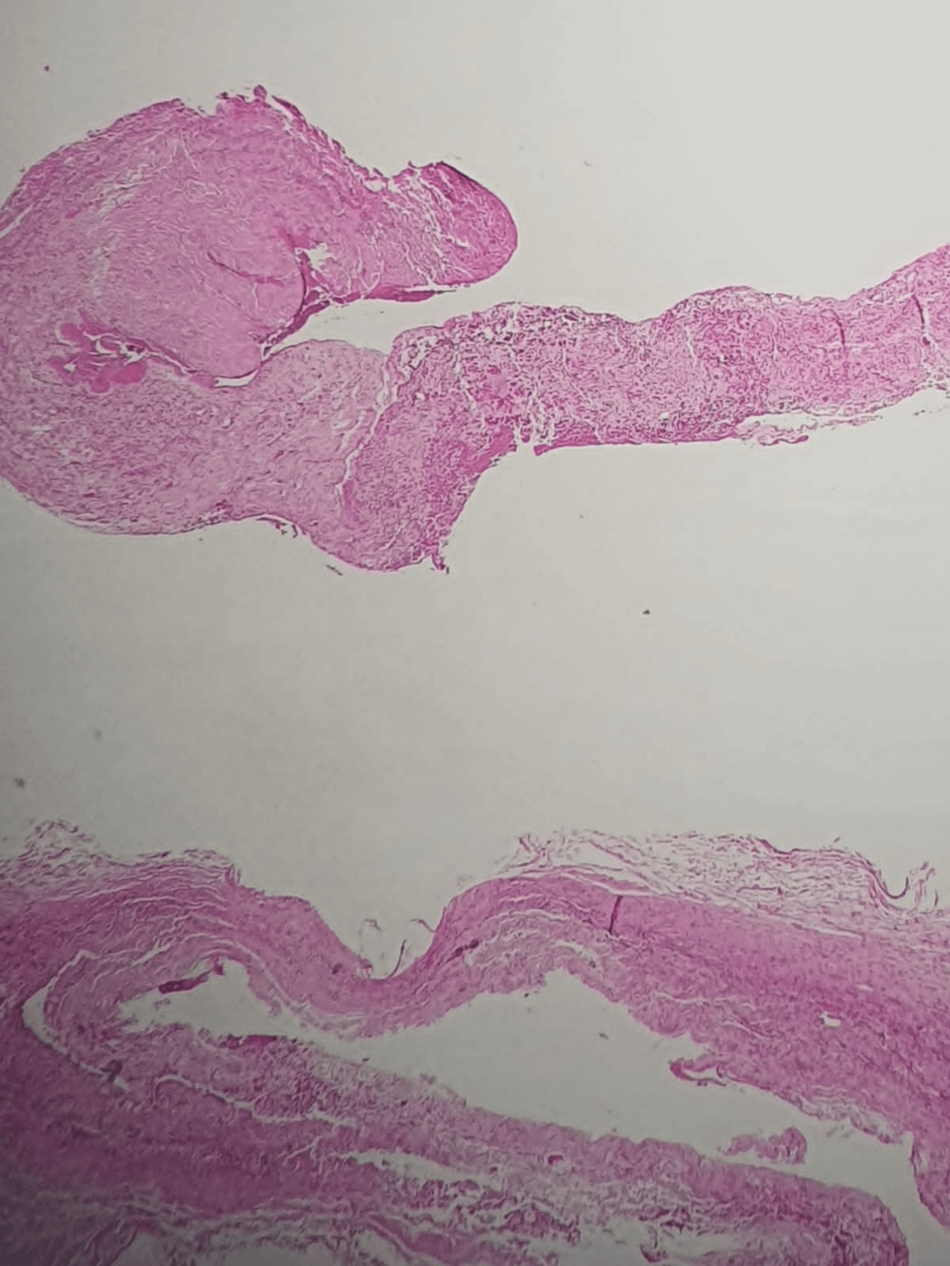
Histopathological photomicrograph showing a capsule of dense fibrous connective tissue with variable thickness, containing delicate blood vessels with extravasation of red blood cells and a discrete inflammatory infiltrate of mononuclear cells, composed of lymphocytes without formation of aggregates. A nest of meningothelial cells and fibrin focally probes the capsule (hematoxylin and eosin, x40).

The cerebrospinal fluid was analyzed for biochemical parameters, viral immunoreactivity, fungal, and bacterial pathogens, and all results were normal. Histopathological examination confirmed the presence of arachnoiditis, characterized by meningothelial cells, dense fibrous tissue, extravasated erythrocytes, and mild lymphocytic inflammation (Figure 4).

**Figure 5:**
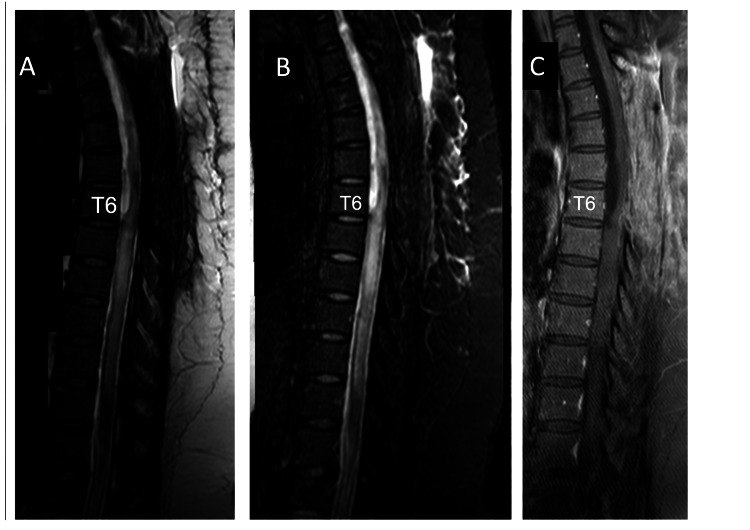
Sagittal T2-weighted (A), T2-STIR (B), and T1-weighted (C) MRI, taken one month after the first decompressive laminectomy from T3 to T6 levels, show a reduction of cystic compression and the presence of spinal cord edema from T6 to T11 levels.

One month after surgery, the T2-weighted (Figure 5A), T2-STIR (Figure 5B), and T1-weighted (Figure 5C) MRI showed a reduction of cystic compression (T3-T6) and the presence of T6-T11 edema.

After an initial improvement, four months later the symptoms recurred, and a new MRI revealed cystic compression again (T3-T6), and the presence of T6-T11 edema.

**Figure 6:**
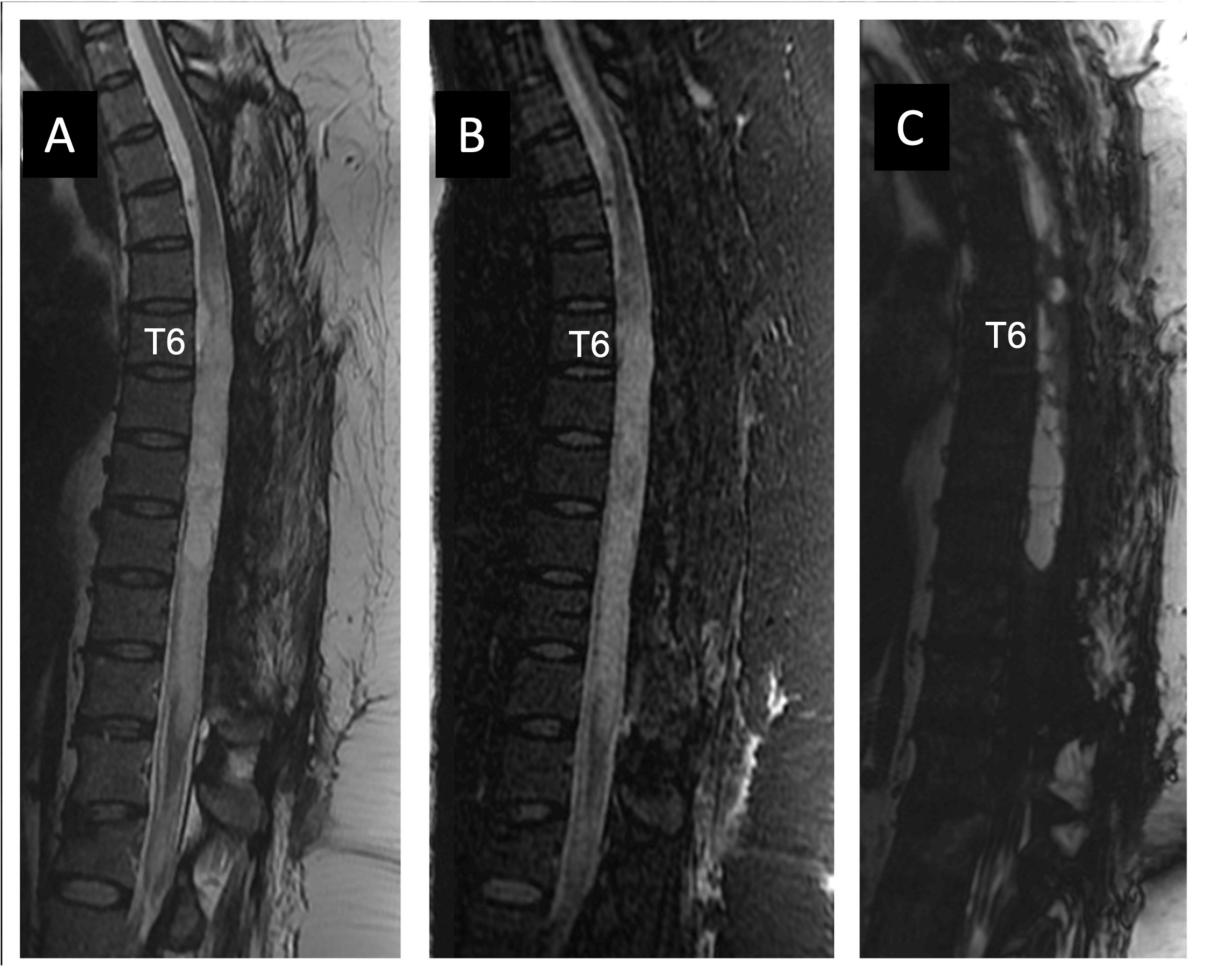
Sagittal T2 (A), T2-STIR (B), and T2-3D (C) MRI, four months after the extended laminectomy from T7 to T11 levels, shows signs of arachnoiditis with adhesions, septations within the dural sac, distortion and displacement of the thoracic spinal cord, and areas of cerebrospinal fluid encystation.

At this moment, a laminectomy was performed from T7 to T11, followed by the opening of the scar tissue from arachnoiditis that involves the spinal cord, and further arachnoid cyst fenestration, allowing for CSF circulation. Despite surgical intervention, subsequent MRI studies conducted four months later showed persistent arachnoiditis, septations, and distortion of the spinal cord (Figure 6A, 6B, 6C). After 18 months of follow-up, the patient shows partial recovery of sensory and motor functions in her lower limbs but remains wheelchair-dependent.

## Discussion

AA is a challenging condition marked by severe inflammation and fibrosis of the arachnoid membranes, leading to neural adhesions and debilitating neurological impairments [6,7]. The inflammatory mechanism and clinical progression characteristic of AA were first documented by Charles Burton in 1970 [7]. Whether idiopathic or resulting from identifiable causes, AA begins with damage and subsequent inflammation of the pia-arachnoid membrane [6]. This inflammatory reaction advances toward granulomatous tissue development, fibrotic changes, and the eventual formation of adhesions [1]. However, the genetic predisposition to aberrant fibrinolytic scarring pathways in the context of AA remains uncertain [1]. The actual prevalence of AA remains unknown, and its occurrence is likely underestimated due to overlooked subclinical cases or instances where AA is not identified as the cause of paraparesis, particularly when spinal canal stenosis is mistakenly attributed to other undiagnosed etiologies [8,9]. A comprehensive literature review identified 12 cases of AA related to anesthesia and obstetric procedures [3-5,10-18]. The clinical characteristics and outcomes of these cases are summarized in Table 1.

**Table 1:** Reported cases of adhesive arachnoiditis following obstetric anesthesia in the literature.

Author (year)	Age	Type of block	First symptom	Onset of symptoms	When was the MRI done?	Treatment	Outcome
Reisner et al. (1980) [4]	33	Epidural	Tetraplegia, headache, and mechanical ventilation assistance.	During the procedure, being discharged 4 weeks after with intermittent bladder catheterization and sensory loss in the lower limbs. Three weeks later, she was readmitted with back pain and loss of lower function.	NA	Steroids	Paresis of lower limbs, sensory abnormalities, urinary dysfunction and fecal incontinence.
Haisa et al (1995) [13]	30	Spinal anesthesia	Sudden sharp painful sensation radiating down to the left leg, and suffered from muscle spasms from the lower back to the calf	At the time of the lumbar puncture	5-6 months	Surgery	The bladder bowel disturbance almost disappeared 3 weeks following surgery, but the pain in the left leg recurred.
Tseng and Lin (1997) [17]	36	Epidural	Numbness and weakness of both lower limbs; urinary retention	On day 1, the patient suffered a shooting pain in the right lower leg during the procedure.	On the same day of the procedure, no abnormalities. 4 months after: arachnoiditis with multiple subarachnoid cysts	Surgery	Motor function improved after surgery, and she could walk without support after a one-year follow-up. However, sphincter and sensory abnormalities persisted.
Garcia and Flores (1999) [12]	15	Epidural	Lower limb Hypoesthesia	12 hours after the epidural	48 hours after the procedure	Steroids/antioxidants	No recurrences and no sequels.
Chiapparini et al. (2000) [11]	34	Epidural	Paraparesis, sensor, and sphincter dysfunction	Hours after the procedure	4 days after the procedure	Surgery	Almost complete recovery.
Ploteau et al. (2004) [3]	30	Epidural	Lower limb weakness and loss of light touch sensation	Five months later but the patient suffered a sudden, sharp, painful sensation radiating to the lower limbs during the procedure	8 years after the procedure	Surgery	Symptoms persisted, and the neurological state of the patient continued to worsen.
Mohamed et al. (2018) [15]	32	Epidural	Asymmetric proximal lower limb weakness	Symptoms were detectable by day 3, but the patient suffered immediate severe back pain during the attempt at the epidural	3 days after: no abnormalities. 27 days after the procedure: signs of adhesion	Steroids	Urinary retention, necessitated catheterization, constipation; after two years, partial upper limb movement was recovered. The patient lost all lower limb strength and could mobilize only with the aid of an electric wheelchair.
Hirai et al. (2012) [14]	29	Combined spinal-epidural	Continuous numbness bilateral lower extremities	1 day after the epidural	5 months after the procedure	Surgery	Three years after the shunt operation, the patient could walk without a cane, although numbness and motor weakness in the lower extremities remained.
Killeen et al. (2012) [18]	27	Spinal	Burning back pain radiating to the legs bilaterally	A few seconds after bupivacaine was administered	11 days after the procedure	Surgery	After 21 months of surgery, the patient had no power in the lower limbs and reduced sensation up to T6 on the left and T10 on the right.
Iga et al. (2019) [5]	40	Combined spinal-epidural	Bilateral lumbociatalgia (S1)	18 hours after the procedure, but the patient suffered radiating pain in her right leg during the injection of the needle	5 days after the procedure	Epidural blood patch	Symptoms improved; the patient referred only to occasional discomfort in the right posterior thigh.
Shimizu (2022) [16]	29	Spinal	Sensory numbness at the left lower leg and weakness ot the left foot.	One day after the procedure	Postoperative day 3	No treatment	Her symptoms gradually improved and entirely disappeared within 2 months.
AlMutairi et al (2024) [10]	33	Epidural	Headache and vomiting	Several weeks later, but five hours after the procedure, she had difficulty standing that was resolved upon discharge.	When the symptoms started, several weeks after the procedure	Ventriculoperitoneal shunt insertion	Symptoms improved entirely.
Fagundes et al. (present case)	39	Spinal	Lower limb weakness, paresthesia at the thoracic spinal level, and decreased light touch and pinprick sensation.	3 months after the procedure, but the patient suffered severe headaches for 23 days after labor	3 months after the procedure	Steroids, acetazolamide, and Surgery	Showed improvement in light touch and lower limb movement two months after the surgery, but still needs the aid of a wheelchair to mobilize.

Although the medical history and physical examination may suggest spinal canal stenosis, MRI analysis can differentiate these conditions by considering the distinct findings observed in each disease. Although non-specific, MRI findings such as nerve root thickening, spinal cord compression, syringomyelia, or arachnoid cysts can suggest impaired cerebrospinal fluid (CSF) dynamics and support the diagnosis of AA [5,8,9]. Subdural arachnoid cysts can sometimes mimic the appearance of AA on MRI, a phenomenon known as the "fake arachnoiditis sign" [9]. This occurs when the cyst entraps the cauda equina, creating an image that resembles nerve root thickening and clumping in AA. Studies have established an association between AA and spinal anesthesia [6,10-20]. Potential contributing factors to this condition include direct nerve injury, adverse reactions to anesthetic drugs or antiseptic agents, and procedural contamination [8,18-20]

Additionally, opioids and steroids have been implicated [1,3]. It is essential to recognize that spinal anesthesia itself can pose a risk factor due to local trauma. The authors emphasized the risk of hazardous injectable fluids like chlorhexidine in the procedural setup and the importance of rigorous contamination prevention measures [16]. Detergents are highly neurotoxic, and their use in equipment for mixing and injecting spinal anesthetic agents should be avoided [16]. Close follow-up is crucial, especially for patients experiencing headaches or sharp pain during or after the procedure. Epidural anesthesia is more frequently associated with AA [6]. An early MRI and corticosteroid therapy initiation at the onset of symptoms led to a favorable outcome [12,13]. Hirai et al. (2012) reported a case in which a patient experienced numbness and weakness in the lower limbs one day after a cesarean section with combined spinal anesthesia. However, the MRI study had a five-month delay [14]. In the present case, symptoms of spinal cord compression appeared later than typically reported in the literature.

Nevertheless, the prolonged headache experienced by the patient after the procedure is consistent with earlier case reports, underscoring the risks associated with delayed MRI evaluation and late recognition of AA [5,10-20]. Most patients reported pain either during the epidural procedure or the following day. Only four cases promptly ordered an MRI upon suspicion of arachnoiditis due to the epidural [12,16,19,20]. The initial MRI showed no abnormalities in two instances, with adhesions only appearing weeks later [16,20]. Regarding treatment, early initiation of steroids has been associated with notable clinical improvement; however, effectiveness depends on the severity of the condition and individual patient responses [19]. Hirai et al. (2012) reported successful symptom management through arachnoid cyst shunting after two ineffective laminectomies [14]. Thecaloscopy and subarachnoepidurostomy, through direct visualization and dissection of the adhesions, have been proposed as less invasive treatment options for AA, arachnoid cysts, and syringomyelia [20]. Ventriculoperitoneal shunts have been reported as a treatment option with successful recovery [9,10,20].

In our case, the patient developed neurologic symptoms three months after a cesarean section performed under spinal anesthesia. While antiseptic skin preparation was unremarkable, the procedure involved multiple lumbar puncture attempts, which may have led to localized trauma or inadvertent exposure to neurotoxic agents. Although neurological symptoms appeared three months after the procedure, earlier clinical suspicion and thorough investigation, particularly given her persistent postpartum headache, could have facilitated prompt steroid initiation and potentially improved outcomes.

## Conclusions

AA is an uncommon yet significant differential diagnosis to consider in patients presenting with progressive back pain, accompanied by sensory or motor deficits in the lower limbs following spinal anesthesia. Although several factors associated with anesthetic procedures have been implicated in its development, the precise pathophysiological mechanisms underlying AA remain poorly understood. Prompt diagnosis using MRI is vital, as imaging findings, while not always pathognomonic, can strongly support the diagnosis when correlated with clinical symptoms. Importantly, even if initial MRIs appear normal shortly after the procedure, continued monitoring is necessary due to the potential for delayed adhesion development. The primary therapeutic approach includes microsurgical decompression of the spinal cord and nerve roots. Additionally, early initiation of corticosteroid therapy upon initial suspicion has demonstrated improved clinical outcomes. Shunting procedures for arachnoid cysts have successfully halted symptom progression in selected cases. Despite intervention, many patients may experience long-term disability, emphasizing the importance of early clinical suspicion, corticosterotherapy, and preventive measures during neuraxial anesthesia.
